# Supplementary material for: Starvation-induced cell fusion and heterokaryosis frequently escape imperfect allorecognition systems in an asexual fungal pathogen
Source: BMC Biol. 2021 Aug 24;19:169. doi: 10.1186/s12915-021-01101-5 (PMC8385987; doi:10.1186/s12915-021-01101-5)
Supplement: Supplementary file 1 — Additional file 1.Table S1. List of strains used in this study. [file 12915_2021_1101_MOESM1_ESM.pdf]

Table S1. *Verticillium dahliae* strains used in this study

strain[1]	background (VCG)	genotype[6]	constructed using plasmid(s)
**Analyses of CAT-mediated fusion (Fig. 1) and post-fusion cellular behavior (Fig. 2)**			
PH H1-mCherry (**a**)	PH (2A)	*nit*M, *VdH1-mCherry, neo*^{R}	pVV19
PH H1-sGFP (**b**)	PH (2A)	*nit1*, *NcH1-sgfp, hph*	pMF357
Ls.17 H1-mCherry[2] (**c**)	Ls.17 (2B)	*nit1*, *VdH1-mCherry-ble*^{R}	fusion PCR
Ls.17 H1-mCherry neo	Ls.17 (2B)	*nit1*, *VdH1-mCherry-ble*^{R}, *neo*^{R}	fusion PCR; pBS-gen
Ls.17 H1-sGFP (**d**)	Ls.17 (2B)	*nit*M, *NcH1-sgfp, hph*	pMF357
Cf.38 H1-sGFP (**e**)	Cf.38 (2B[3])	*nit*M, *NcH1-sgfp, hph*	pMF357
115 H1-sGFP (**f**)	115 (2B)	*nit*M, *NcH1-sgfp, hph*	pMF357
BB H1-sGFP (**g**)	BB (4A)	*nit1*, *NcH1-sgfp, hph*	pMF357
Ca.146 H1-sGFP (**h**)	Ca.146 (6)	*nit*M, *NcH1-sgfp, hph*	pMF357
Ca.148 H1-mCherry (**i**)	Ca.148 (6)	*nit1, VdH1-mCherry, neo*^{R}	pVV19
Ls.17 sGFP[2]	Ls.17 (2B)	*sgfp*, *hph*	pIGPAPA
Cf.38 sGFP	Cf.38 (2B[5])	*sgfp*, *hph*	pIGPAPA
PH sGFP	PH (2A)	*sgfp*, *hph*	pIGPAPA
**Complementation assays (Fig. 3)**			
T9.M[3] (**A**)	T9 (1A)	*nit*M	–
V607I.M[3] (**B**)	V607I (1B)	*nit*M	–
123V.M[2] (**C**)	123V (2A)	*nit*M	–
115.M[3] (**D**)	115 (2B)	*nit*M	–
Cf.38.M[3] (**E**)	Cf.38 (2B[5])	*nit*M	–
PCW.M[3] (**F**)	PCW (3)	*nit*M	–
Dvd-E6.M[3] (**G**)	Dvd-E6 (4A)	*nit*M	–
S39.M[3] (**H**)	S39 (4B)	*nit*M	–
Ca.146.M[3] (**I**)	Ca.146 (6)	*nit*M	–
V13.M[3] (**J**)	V13 (HSI[4])	*nit*M	–
Ms.102.M[3] (**K**)	*V. nonalfalfae* Ms.102	*nit*M	–
V44.1[3] (**L**)	V44 (1A)	*nit1*	–
V661I.1[3] (**M**)	V661I (1B)	*nit1*	–
PH.1[3] (**N**)	PH (2A)	*nit1*	–
Ls.17.1[3] (**O**)	Ls.17 (2B)	*nit1*	–
70-21.1[3] (**P**)	70-21 (3)	*nit1*	–

BB.1[3] (**Q**)	BB (4A)	*nit1*	–
V684I.1[3] (**R**)	V684I (4B)	*nit1*	–
Ca.148.1[3] (**S**)	Ca.148 (6)	*nit1*	–
Cf.162.1[3] (**T**)	Cf.162 (HSI[4,5])	*nit1*	–
T2.1[3] (**U**)	*V. nonalfalfae* T2	*nit1*	–
**Mixed infection experiments**			
Ls.17 *neo*^{R}	Ls.17 (2B)	*neo*^{R}	pBS-genR
Ls.17 *hph*	Ls.17 (2B)	*hph*	pUCATPH
Cf.38 *hph*	Cf.38 (2B[5])	*hph*	pUCATPH
PH *hph*	PH (2A)	*hph*	pUCATPH
BB *hph*	BB (4A)	*hph*	pUCATPH
Ca.146 *hph*	Ca.146 (6)	*hph*	pUCATPH
**Analyses of autophagic genes**			
Ls.17 mCherry-atg8	Ls.17 (2B)	*mCherry-atg8*	pVV27; pVV25
Ls.17 H1-mChery sGFP-atg8	Ls.17 H1-mCherry neo	*nit1*, *VdH1-mCherry-ble*^{R}, *neo*^{R}, *sGFP-atg8*	pVV27; pVV26
Ls.17-Δatg1	Ls.17 H1-mCherry neo	*nit1*, *VdH1-mCherry-ble*^{R}, *neo*^{R}, Δ*atg1*::*hph*	pOSCAR-atg1
Ls.17-Δatg8	Ls.17 H1-mCherry	*nit1, VdH1-mCherry-ble*^{R}, *atg8::neo*^{R}	pVV27; 5'H_{atg8}-*neo*^{R}-3'H_{atg8} (H: 100 bp-long homology arms)
PH-Δatg1	PH (2A)	Δ*atg1::hph*	pOSCAR-atg1
Ls.17-Δatg1-c	Ls.17-Δatg1	Δ*atg1*::*hph*, *neo*^{R}	pBS-genR, wild-type *atg1*
Ls.17-Δatg8-c	Ls.17-Δatg8	*atg8*:: *neo*^{R}, *hph*	pUCATPH, wild-type *atg8*

[1] Single-letter codes (in brackets) correspond to the strains shown in Fig. 1 and 3.

[2] Reference: [37].

[3] Reference: [36].

[4] HSI: Heterokaryon Self-Incompatible.

[5] Isolates Cf.38 and Cf.162 were originally classified into VCG 6, but their classification was later re-assessed [38].

[6] *Vd: Verticillium dahliae*; *Nc: Neurospora crassa*.
