# Supplementary material for: Starvation-induced cell fusion and heterokaryosis frequently escape imperfect allorecognition systems in an asexual fungal pathogen
Source: BMC Biol. 2021 Aug 24;19:169. doi: 10.1186/s12915-021-01101-5 (PMC8385987; doi:10.1186/s12915-021-01101-5)
Supplement: Supplementary file 2 — Additional file 2.Table S2. Results of time-lapse imaging experiments. [file 12915_2021_1101_MOESM2_ESM.pdf]

Table S2. Cell fate and nuclear behavior (types I-IV, details in the text) of *V. dahliae* conidia/germlings involved in CAT-mediated fusion (summarized in Fig. 2)

pairing	type				cytoplasmic flow		total
	I	II	III	IV	yes	no	
**self**							
Ls.17-H1-mCherry × Ls.17-H1-sGFP	150	92	4	0	-	-	246
PH-H1-mCherry × PH-H1-sGFP	14	9	0	0	-	-	23
Ls.17-sGFP × Ls.17-H1-sGFP	28	7	0	0	35	0	35
**“compatible” (i.e., same VCG)**							
Ls.17-H1-mCherry × 115-H1-sGFP	12	0	7	2	-	-	21
Ls.17-H1-mCherry × Cf.38-H1-sGFP	20	0	15	1	-	-	36
Ca.148-H1-mCherry × Ca.146-H1-sGFP	11	0	6	0	-	-	17
Ls.17-sGFP × Cf.38-H1-sGFP	6	0	0	0	6	0	6
**“incompatible” (i.e., different VCGs)**							
Ls.17-H1-mCherry × PH-H1-sGFP	97	0	14	42	-	-	153
Ls.17-H1-mCherry × BB-H1-sGFP	91	0	23	198	-	-	312
Ls.17-H1-mCherry × Ca.146-H1-sGFP	12	0	2	23	-	-	37
PH- sGFP × BB-H1-sGFP	10	0	0	12	14	8	22
