# Supplementary material for: Starvation-induced cell fusion and heterokaryosis frequently escape imperfect allorecognition systems in an asexual fungal pathogen
Source: BMC Biol. 2021 Aug 24;19:169. doi: 10.1186/s12915-021-01101-5 (PMC8385987; doi:10.1186/s12915-021-01101-5)
Supplement: Supplementary file 4 — Additional file 4 Supplementary Figures. Figure S1: Typical features of incompatibility-triggered cell death in V. dahliae. Figure S2: Fluorescent tagging of V. dahliae Atg8 by a CRISPR/Cas9-based system. Figure S3: Construction and validation of V. dahliae atg1 and atg8 knockout mutants. Figure S4: Atg8-mediated nuclear degradation. Figure S5: Frequencies of multinucleate cells in complemented Δatg1-c and Δatg8-c strains. [file 12915_2021_1101_MOESM4_ESM.pdf]

Supplementary Figures

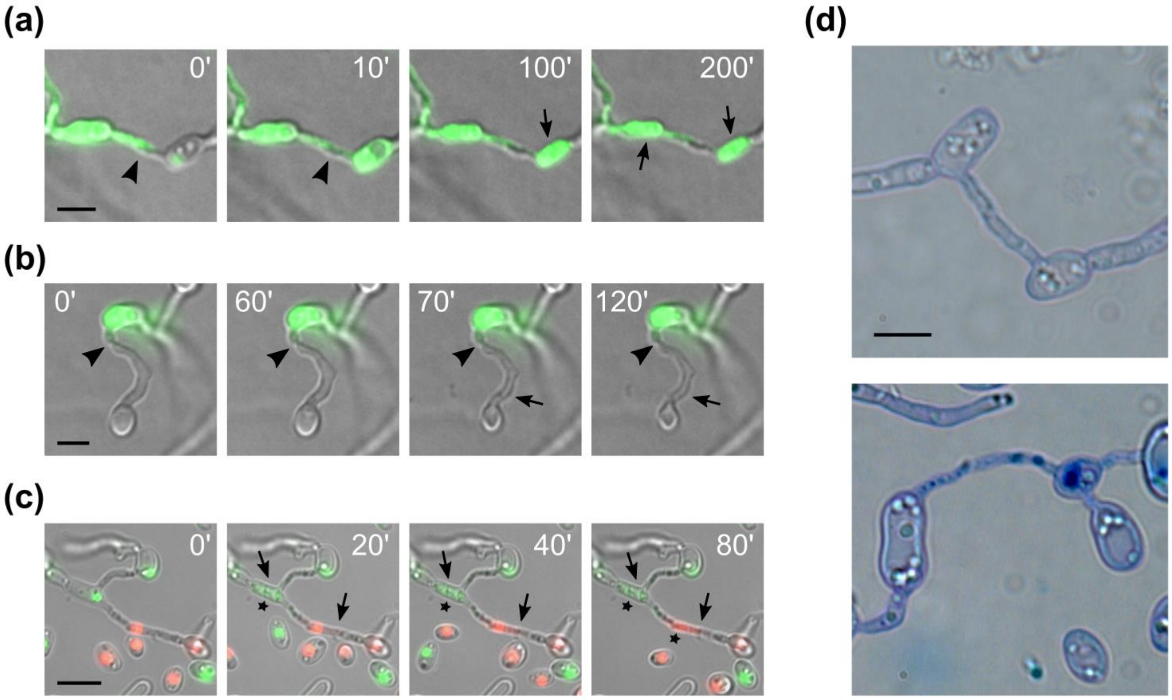

Fig. S1. Typical features of incompatibility-triggered cell death in *V. dahliae*. **a** Induction of an incompatibility reaction after cytoplasmic mixing. Strains: PH sGFP and BB H1-sGFP. **b** Induction of an incompatibility reaction without prior cytoplasmic mixing. Strains: PH sGFP and BB. **c** Incompatibility-triggered cell death is characterized by nuclear degradation and cell shrinkage. Strains: PH H1-sGFP and Ls.17 H1-mCherry. In **a**-**c** arrowheads indicate the contact point of germlings, and arrows indicate cell shrinkage. **d** Staining of CAT-mediated fused cells using methylene blue. Viable fusions remain unstained (top), whereas post-fusion cell death permits the accumulation of the dye in the cytoplasm (bottom). Bars = 5 μm

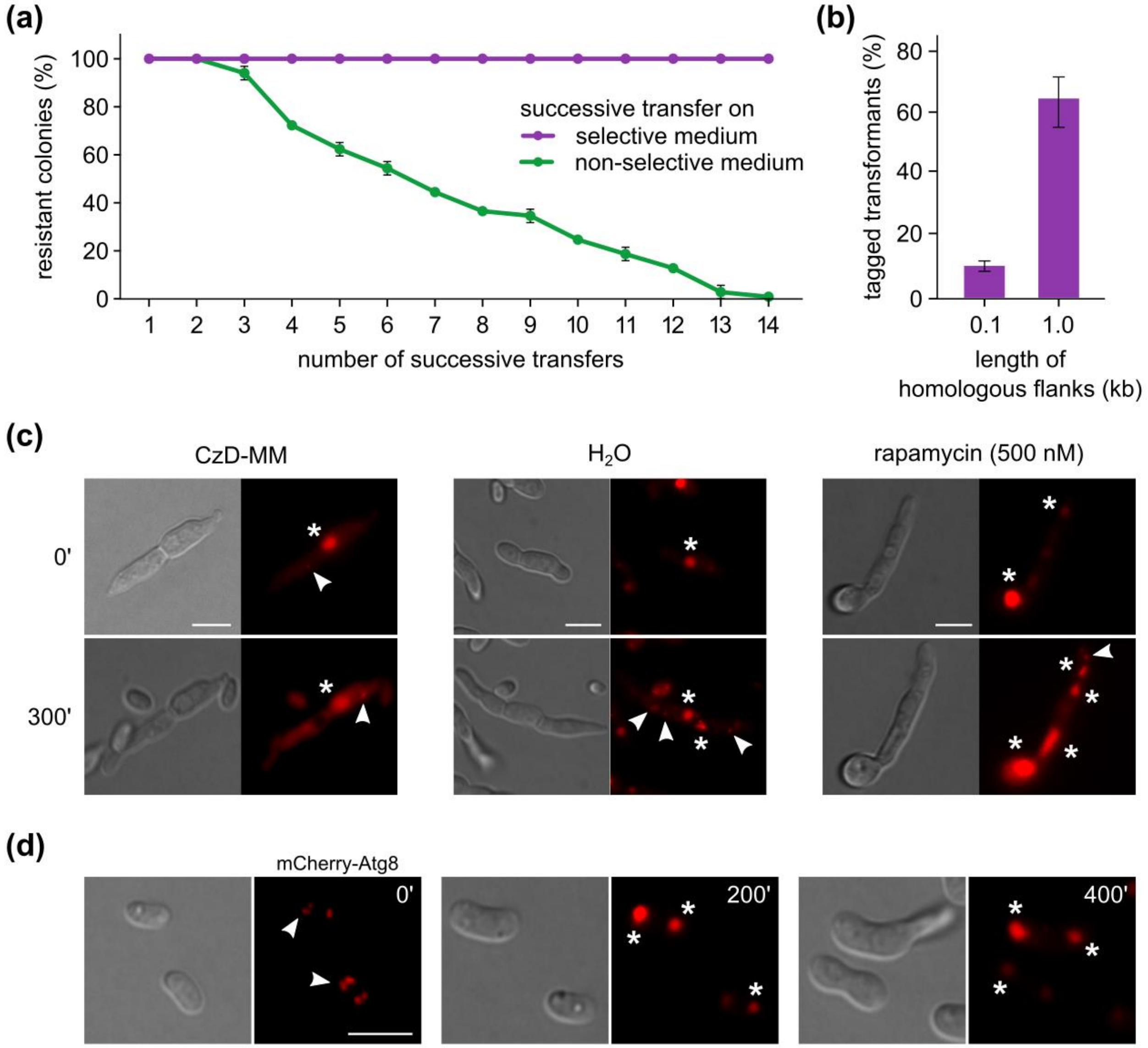

Fig. S2. Fluorescent tagging of *V. dahliae* Atg8 by a CRISPR/Cas9-based system. **a** Stability of AMA1-containing plasmids under selective and non-selective conditions. Twenty-five randomly selected transformants of *V. dahliae* Ls.17 were single-spore purified and re-cultured every three days on selective (PDA supplemented with hygromycin B) and non-selective (PDA) medium. After each transfer, the percentage of resistant colonies to hygromycin B was determined. The experiment was performed in triplicate. Bars = SD. **b** Tagging efficiency of Atg8 with mCherry (strain Ls.17), using repair substrates with different lengths of homologous arms. The use of 0.1 kb-long arms yielded mostly chimeric fluorescent colonies consisting of tagged and non-tagged cell populations (13% properly tagged cells on average), in contrast to 1.0 kb-long arms that resulted in more homogeneous colonies (i.e., at least 90% of cells were

properly tagged). The experiment was performed in triplicate for each repair construct, and at least 20 independent transformants were checked per replicate (using PCR and microscopy). Bars = SD. **c** Effect of autophagy-inducing conditions on the mCherry-Atg8 localization. Both starvation (i.e., incubation in water) and treatment with rapamycin (i.e., a TOR kinase inhibitor that induces autophagy) lead to the accumulation of the protein in autophagosomes and vacuoles, which indicates the induction of autophagy. **d** Subcellular localization of mCherry-Atg8 during germination in minimal medium. Prior to germination, the protein forms small globular foci (presumably autophagosomes, arrowheads), which seem to accumulate during germination in larger structures (vacuoles, asterisks), which is consistent with the expected participation of autophagy in conidial germination

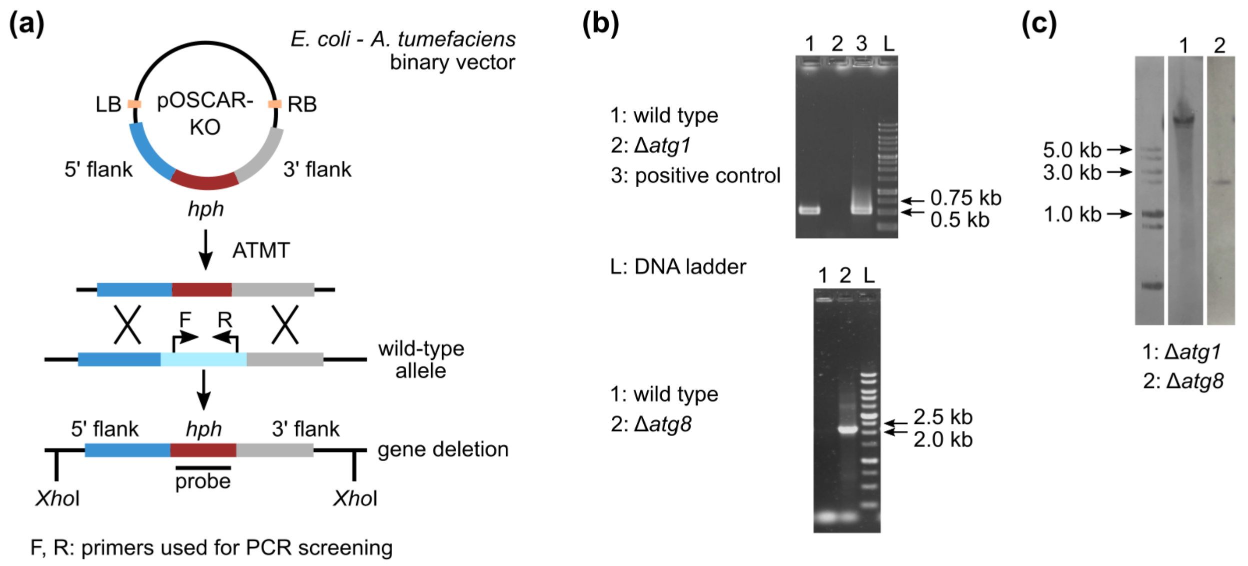

Fig. S3. Construction and validation of *V. dahliae atg1* and *atg8* knockout mutants. **a** Schematic representation of the double homologous recombination-based strategy using *Agrobacterium tumefaciens*-mediated transformation for deletion of *atg1*. **b** Mutant validation by PCR using gene-specific (internal) primers (i.e., Vdatg1F/R) for the deletion of *atg1* and the combination of a gene-specific primer with a primer binding to the selection marker (i.e., atg8-gen-F/3flatg8R) for the CRISPR/Cas9-mediated disruption of *atg8*. **c** Validation of mutants as single integration events by Southern hybridization with the corresponding labeled selection marker cassettes as probes (i.e., *hph* for *atg1*, *neo*^{R} for *atg8*; genomic DNA of all strains was digested by *Xho*I)

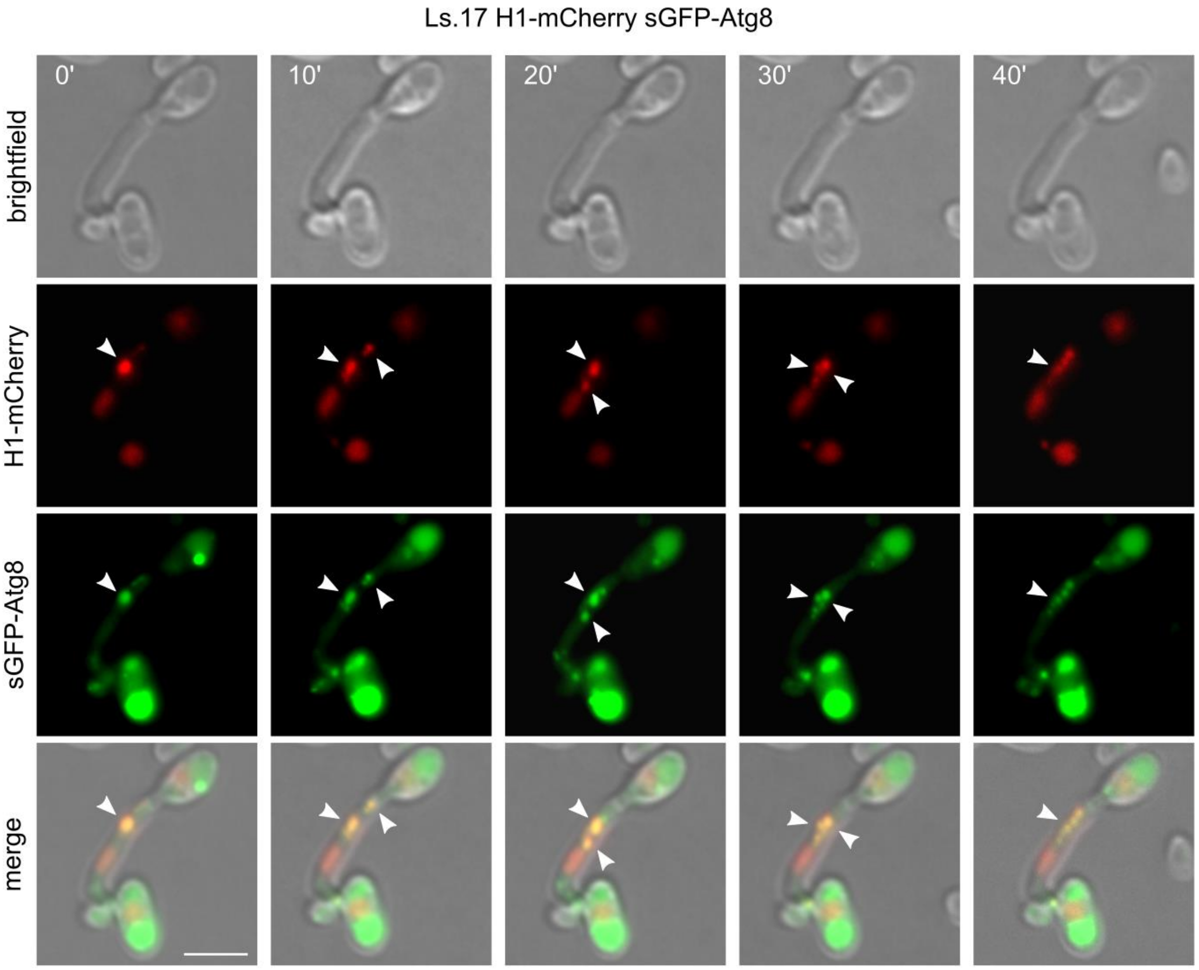

Fig. S4. Atg8-mediated nuclear degradation in the strain Ls.17 H1-mCherry sGFP-Atg8. Atg8 is co-localized with the degrading nucleus during this process (arrowheads). Bars = 5 μm

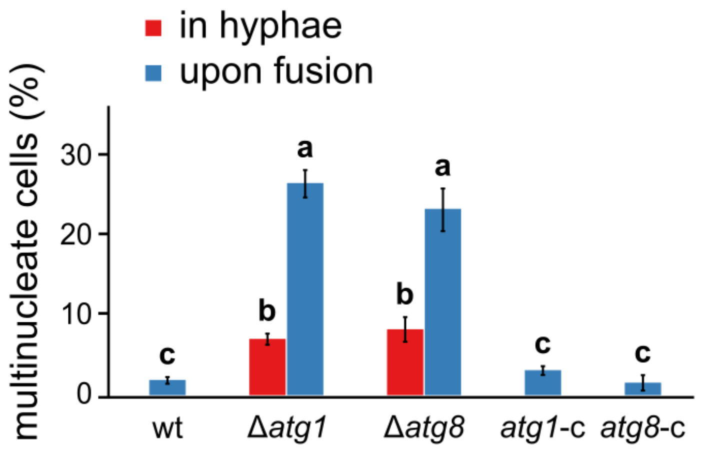

Fig. S5. Frequencies of non-apical hyphal compartments and fused cells with more than one nuclei in complemented Δ*atg1* and Δ*atg8* knockout strains (by re-introducing the corresponding wild-type genes) in strain *V. dahliae* Ls.17. Each strain was tested in triplicate (n = 300 hyphal cells or 150 anastomoses per replicate). Statistical significance of differences was tested with one-way ANOVA followed by Tukey's post-hoc test; bars with the same letter do not differ significantly (p value > 0.05)
