# Supplementary material for: Starvation-induced cell fusion and heterokaryosis frequently escape imperfect allorecognition systems in an asexual fungal pathogen
Source: BMC Biol. 2021 Aug 24;19:169. doi: 10.1186/s12915-021-01101-5 (PMC8385987; doi:10.1186/s12915-021-01101-5)
Supplement: Supplementary file 6 — Additional file 6.Table S3. Results of complementation assays. [file 12915_2021_1101_MOESM6_ESM.pdf]

Table S3. Results of the complementation tests summarized in Fig. 3. The vigor of each interaction is indicated by "+" (i.e., strong reaction characterized by dense prototrophic mycelial growth and extended production of microsclerotia), "+/-" (i.e., weak reaction with limited mycelial growth and/or restricted production of microsclerotia) or "-" (i.e., absence of prototrophic growth). Each pairing was performed in at least three replicates. Pairings that produced very limited prototrophic growth in only one of the replicates were regarded as negative ("-")

pairing	VCGs	assay	
		hyphal-based / VCG (Fig. 3a, b)	CAT-based (Fig. 3c, d)
T9.M × V44.1	1A × 1A	+	+
T9.M × V661I.1	1A × 1B	+/-	+
T9.M × PH.1	1A × 2A	-	+/-
T9.M × Ls.17.1	1A × 2B	-	-
T9.M × 70-21.1	1A × 3	-	+/-
T9.M × BB.1	1A × 4A	-	+/-
T9.M × V684I.1	1A × 4B	-	+/-
T9.M × Ca.148.1	1A × 6	-	+/-
T9.M × Cf.162.1	1A × HSI	-	-
T9.M × T2.1	1A × *V. nonalfalfae*	-	+/-
V607I.M × V44.1	1B × 1A	-	+/-
V607I.M × V661I.1	1B × 1B	+/-	+/-
V607I.M × PH.1	1B × 2A	-	-
V607I.M × Ls.17.1	1B × 2B	-	-
V607I.M × 70-21.1	1B × 3	-	+/-
V607I.M × BB.1	1B × 4A	-	+/-
V607I.M × V684I.1	1B × 4B	-	-
V607I.M × Ca.148.1	1B × 6	-	+/-
V607I.M × Cf.162.1	1B × HSI	-	+/-
V607I.M × T2.1	1B × *V. nonalfalfae*	-	-
123V.M × V44.1	2A × 1A	-	-
123V.M × V661I.1	2A × 1B	-	+/-
123V.M × PH.1	2A × 2A	+	+
123V.M × Ls.17.1	2A × 2B	-	+
123V.M × 70-21.1	2A × 3	-	-
123V.M × BB.1	2A × 4A	-	+/-

123V.M × V684I.1	2A × 4B	-	-
123V.M × Ca.148.1	2A × 6	-	+/-
123V.M × Cf.162.1	2A × HSI	-	-
123V.M × T2.1	2A × *V. nonalfalfae*	-	+/-
115.M × V44.1	2B × 1A	-	-
115.M × V661I.1	2B × 1B	-	+/-
115.M × PH.1	2B × 2A	-	+/-
115.M × Ls.17.1	2B × 2B	+/-	+
115.M × 70-21.1	2B × 3	-	+/-
115.M × BB.1	2B × 4A	-	+/-
115.M × V684I.1	2B × 4B	-	+/-
115.M × Ca.148.1	2B × 6	-	+/-
115.M × Cf.162.1	2B × HSI	-	+/-
115.M × T2.1	2B × *V. nonalfalfae*	-	-
Cf.38.M × V44.1	2B × 1A	-	-
Cf.38.M × V661I.1	2B × 1B	-	-
Cf.38.M × PH.1	2B × 2A	-	+/-
Cf.38.M × Ls.17.1	2B × 2B	+	+
Cf.38.M × 70-21.1	2B × 3	-	+/-
Cf.38.M × BB.1	2B × 4A	-	+/-
Cf.38.M × V684I.1	2B × 4B	-	+/-
Cf.38.M × Ca.148.1	2B × 6	-	+/-
Cf.38.M × Cf.162.1	2B × HSI	-	-
Cf.38.M × T2.1	2B × *V. nonalfalfae*	-	-
Dvd-E6.M × V44.1	3 × 1A	-	-
Dvd-E6.M × V661I.1	3 × 1B	-	+/-
Dvd-E6.M × PH.1	3 × 2A	-	-
Dvd-E6.M × Ls.17.1	3 × 2B	-	+/-
Dvd-E6.M × 70-21.1	3 × 3	+	+/-
Dvd-E6.M × BB.1	3 × 4A	-	+/-
Dvd-E6.M × V684I.1	3 × 4B	+/-	+/-
Dvd-E6.M × Ca.148.1	3 × 6	-	-
Dvd-E6.M × Cf.162.1	3 × HSI	-	+/-
Dvd-E6.M × T2.1	3 × *V. nonalfalfae*	-	-
PCW.M × V44.1	4A × 1A	-	+/-
PCW.M × V661I.1	4A × 1B	-	+/-

PCW.M × PH.1	4A × 2A	-	-
PCW.M × Ls.17.1	4A × 2B	-	-
PCW.M × 70-21.1	4A × 3	-	+
PCW.M × BB.1	4A × 4A	+	+/-
PCW.M × V684I.1	4A × 4B	-	+
PCW.M × Ca.148.1	4A × 6	-	+/-
PCW.M × Cf.162.1	4A × HSI	-	+/-
PCW.M × T2.1	4A × *V. nonalfalfae*	-	+/-
S39.M × V44.1	4B × 1A	-	-
S39.M × V661I.1	4B × 1B	-	-
S39.M × PH.1	4B × 2A	-	-
S39.M × Ls.17.1	4B × 2B	-	+/-
S39.M × 70-21.1	4B × 3	-	-
S39.M × BB.1	4B × 4A	-	-
S39.M × V684I.1	4B × 4B	+/-	+/-
S39.M × Ca.148.1	4B × 6	-	+/-
S39.M × Cf.162.1	4B × HSI	-	-
S39.M × T2.1	4B × *V. nonalfalfae*	-	-
Ca.146.M × V44.1	6 × 1A	-	+/-
Ca.146.M × V661I.1	6 × 1B	-	+/-
Ca.146.M × PH.1	6 × 2A	-	-
Ca.146.M × Ls.17.1	6 × 2B	-	-
Ca.146.M × 70-21.1	6 × 3	-	+/-
Ca.146.M × BB.	6 × 4A	-	-
Ca.146.M × V684I.1	6 × 4B	-	+/-
Ca.146.M × Ca.148.1	6 × 6	+	+
Ca.146.M × Cf.162.1	6 × HSI	-	+/-
Ca.146.M × T2.1	6 × *V. nonalfalfae*	-	+/-
V13.M × V44.1	HSI × 1A	-	-
V13.M × V661I.1	HSI × 1B	-	-
V13.M × PH.1	HSI × 2A	-	-
V13.M × Ls.17.1	HSI × 2B	-	-
V13.M × 70-21.1	HSI × 3	-	+/-
V13.M × BB.1	HSI × 4A	-	+/-
V13.M × V684I.1	HSI × 4B	-	-
V13.M × Ca.148.1	HSI × 6	-	-

V13.M × Cf.162.1	HSI × HSI	-	-
V13.M × T2.1	HSI × *V. nonalfalfae*	-	-
Ms.102.M × V44.1	*V. nonalfalfae* × 1A	-	-
Ms.102.M × V661I.1	*V. nonalfalfae* × 1B	-	+/-
Ms.102.M × PH.1	*V. nonalfalfae* × 2A	-	-
Ms.102.M × Ls.17.1	*V. nonalfalfae* × 2B	-	-
Ms.102.M × 70-21.1	*V. nonalfalfae* × 3	-	+/-
Ms.102.M × BB.1	*V. nonalfalfae* × 4A	-	+/-
Ms.102.M × V684I.1	*V. nonalfalfae* × 4B	-	+/-
Ms.102.M × Ca.148.1	*V. nonalfalfae* × 6	-	+/-
Ms.102.M × Cf.162.1	*V. nonalfalfae* × HSI	-	-
Ms.102.M × T2.1	*V. nonalfalfae*	+/-	+/-
