# Supplementary figures and images for: Starvation-induced cell fusion and heterokaryosis frequently escape imperfect allorecognition systems in an asexual fungal pathogen
Source: BMC Biol. 2021 Aug 24;19:169. doi: 10.1186/s12915-021-01101-5 (PMC8385987; doi:10.1186/s12915-021-01101-5)

**Table S4.** *Verticillium dahliae* homologs of autophagy genes

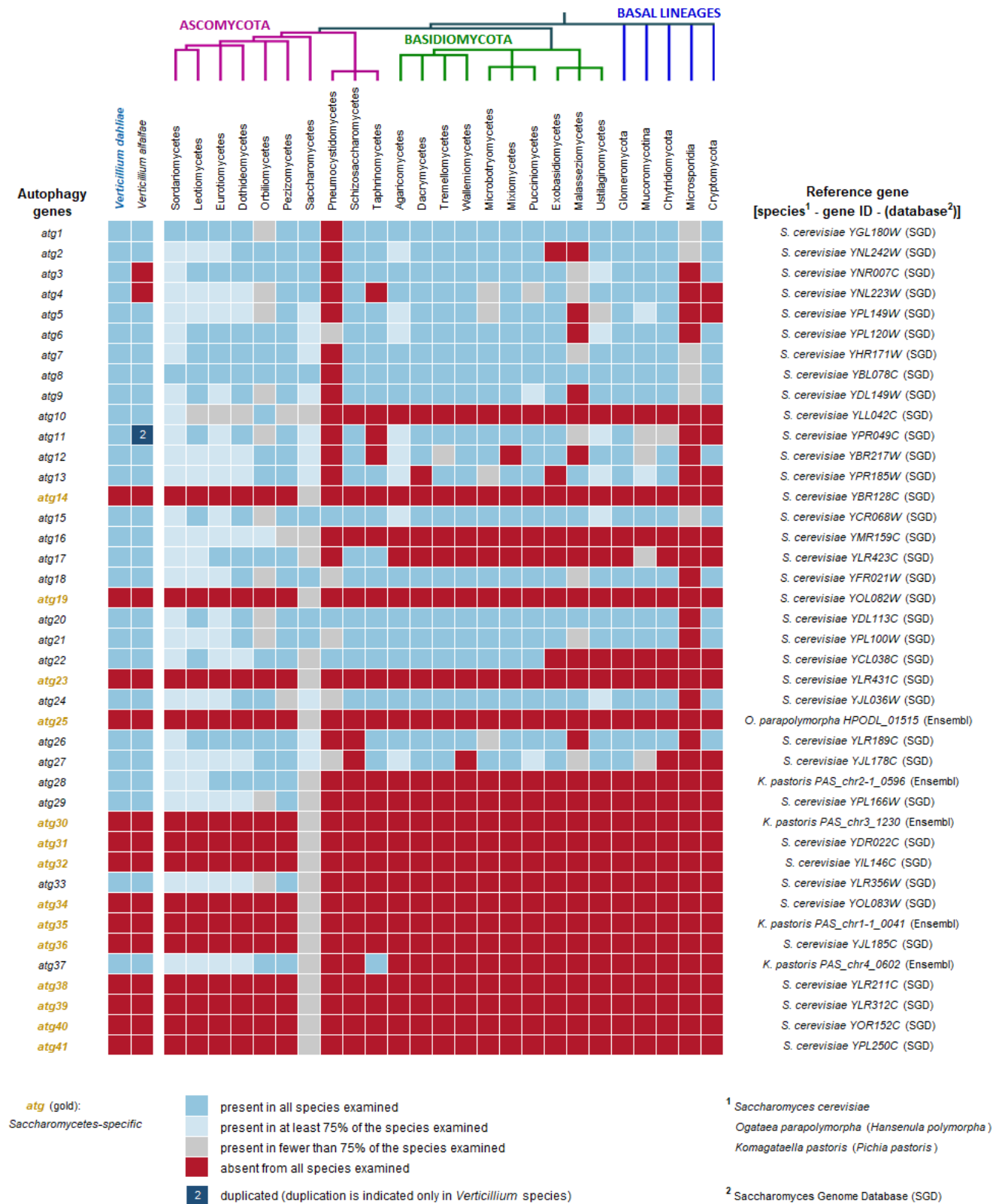

Supplement: Supplementary file 7 — Additional file 7 Table S4. Verticillium dahliae homologs of autophagy genes. [file 12915_2021_1101_MOESM7_ESM.pdf]
