# Supplementary material for: Starvation-induced cell fusion and heterokaryosis frequently escape imperfect allorecognition systems in an asexual fungal pathogen
Source: BMC Biol. 2021 Aug 24;19:169. doi: 10.1186/s12915-021-01101-5 (PMC8385987; doi:10.1186/s12915-021-01101-5)
Supplement: Supplementary file 12 — Additional file 12.Table S5. List of plasmids. [file 12915_2021_1101_MOESM12_ESM.pdf]

Table S5. List of plasmids constructed and used in this study

plasmid	backbone	description	source
		**recombinant vector construction**	
pUCATPH	-	*hph*	[85]
pMaM330	pFA6a	*mCherry-myeGFP-kanMX6*	[86]
pSD1	pBluescript II	P_{gpda}, P_{trpc}, *neo*^{R}	[87]
pBS-genR	pBluescript II	*neo*^{R}	[68]
pFC332	-	AMA1, *hph*, P_{tef1}-*SpCas9*-T*tef1*	[43]
pFC334	-	AMA1, *argB*, P_{gpdA}-HH-sgRNA backbone-HDV -T_{trpC}	[43]
pOSCAR	pPZP-RCS2	*A. tumafaciens* binary vector	[88]
		**fluorescent labeling**	
pMF357	-	P_{ccg1}-*NcH1-sgfp, hph*	[59]
pVV19	pOSCAR	P_{VdH1}-*VdH1-mCherryFP*-T_{tef1}, *neo*^{R}	This study
pIGPAPA	-	P_{ICL}-*sgfp*-T_{nosI}, *hph*	[89]
		**CRISPR/Cas9 mediated *atg*8-targeting**	
pVV25	pOSCAR	5'H_{Vdatg8}-*sgfp*-3'H_{Vdatg8} (H: 1.0 kb-long homology arms)	This study
pVV26	pOSCAR	5'H_{Vdatg8}-*mCherryFP*-3'H_{Vdatg8} (H: 1.0 kb-long homology arms)	This study
pVV27	pFC332	P_{gpdA}-HH-crRNA for *Vdatg8*-HDV-T_{trpC}	This study
		***atg1* deletion**	
pOSCAR-atg1	pOSCAR	5'H_{Vdatg1}-*hph*-3'H_{Vdatg1} (H: 2.0 kb-long homology arms)	This study
