# Supplementary material for: Starvation-induced cell fusion and heterokaryosis frequently escape imperfect allorecognition systems in an asexual fungal pathogen
Source: BMC Biol. 2021 Aug 24;19:169. doi: 10.1186/s12915-021-01101-5 (PMC8385987; doi:10.1186/s12915-021-01101-5)
Supplement: Supplementary file 13 — Additional file 13. Table S6. List of PCR primers. [file 12915_2021_1101_MOESM13_ESM.pdf]

Table S6. List of DNA oligonucleotides used in this study

oligo name	sequence (5' to 3')	template (strain/ plasmid)	construction/ validation of strain/plasmid
	**nuclear labeling with H1-mCherry**		
VdH1F	GAAACGACAATCTGATCCAAGCTCAAGCTACGGGAGACACAGGACATCATG	Ls.17	pVV19
VdH1R	GCCCTTGCTCACCATTGCGGAAGCAGCAGCAGC		
h1tefF	GTACAAGCTCGACTAAGCGGACATTCGATTTATGC	pFC332	
h1tefR	CAATATCAGTTAACGTCGTATTGGGATGAATTTTGTATGC		
chFPF	CTGCTGCTTCCGCAATGGTGAGCAAGGGCGAG	pMaM330	
chFPR	AAATCGAATGTCCGCTTAGTCGAGCTTGTACAGCTCG		
h1genF	CAAAATTCATCCCAATACGACGTTAACTGATATTGAAGGAGCAC	pSD1	
h1genR	CTTTATGCCTGCAGGTCGCGAGCGATCGCGGTACAACCCAGGGCTGGTGACGG		
	**CRISPR/Cas9-mediated *atg8* targeting**		
334-a-F	CATCTGCGGAACATATACTGGGCCCGGGAAGCGTAAGCTCCCTAATTGGC	pFC334	pVV27
334-b-R	TGATTCTGCTGTCTCGGCTGAGGTCTTAATGAGCCAAGAGCGGATTCCTC		
334-a-atg8-R	GACGAGCTTACTCGTTTCGTCCTCACGGACTCATCAGCAGTATCGGTGATGTCTGCTCAAGCG		
334-b-atg8-F	TCCGTGAGGACGAAACGAGTAAGCTCGTCCAGTATGCGATCCAAGTTCAGTTTTAGAGCTAGAAATAGCAAGTTAAA		
5flatg8F	GAAACGACAATCTGATCCAAGCTCAAGCTAAAGGGGACAGCGTTATTATG	Ls.17	pVV25, pVV26
5flatg8R	GCCCTTGCTCACCATGGTTAGGTAGAAGAGGTACG		
3flatg8F	GACGAGCTGTACAAGATGCGATCCAAGTTCAAGG		
3flatg8R	CTTTATGCCTGCAGGTCGCGAGCGATCGCGGTACGATCACAGCCAGTCCAATAC		
gfp-atg8F	CTCTTCTACCTAACCATGGTGAGCAAGGGCGAG	pIGPAPA	pVV25, pVV26
gfp-atg8R	GAACTTGGATCGCATCTTGTACAGCTCGTCCATGCC		
mchFP-atg8F	CTCTTCTACCTAACCATGGTGAGCAAGGGCGAG	pMaM330	
mchFP-atg8R	GAACTTGGATCGCATGTCGAGCTTGTACAGCTCGTC		
5HA-Vdatg8-F	TAAGCCCTTCTGCTTCACCCTTCTCGATCGACGGACCTGTCAACAGCTGCAACGACCCCAACCACAACCGACAGTGCAAACGTACCTCTTCTACCTAACCATGTGGAGCAAGGGCGAG	pIGPAPA, pMaM330	Ls.17-mCheryy-atg8
3HA-Vdatg8-R	CCACATACAGGAATACGGTCCGAGTACTTCTGACGAATGCGCTCGGCCTCAGCCTTACGCTTCTCGAAGGGGTGCTCGTCCTTGAACTTGGATCGCATGTCGAGCTTGTACAGCTCGTC		
atg8-gen-F	CTTCTGCTTCACCCTTCTCGATCGACGGACCTGTCAACAGCTGCAACGACCCCAACCACAACCGACAGTGCAAACGTACCTCTTCTACCTAACCGACGTTAACTGATATTGAAGGAGCAC	pSD1	Ls.17-Δatg8

atg8-gen-R	ACGATGCCCGCCCATGACCACATTTACTCTTCTGACGAATGCGCTCGGCCTCAAGCCTTACGCTTCTCGAAGGGGTGCTAGTCCTTGAACTTGGATCACATAACCCAGGGCTGGTGACGG		
***atg1* deletion**			
5flatg1F	GAAACGACAATCTGATCCAAGCTCAAGCTAGTTATCGCACGCATCCAGC	Ls.17	pOSCAR-atg1
5flatg1R	CCACTAGCATTACACGAACACGGCAGCTTGTAGC		
3flatg1F	GAATAAGGGCGACACAAGTCAACCCATCGCAGGCTG		
3flatg1R	GCCTGCAGGTCGCGAGCGATCGCGGTACCACCGTCTCCTCCTCCA		
atg1hygF	CAAGCTGCCGTGTTCGTGTAATGCTAGTGGAGGTCAAC	pUCATPH	
atg1hygR	CTGCGATGGGTTGACTTGTGTCGCCCTTATTCGAC		
Vdatg1F	GCGGATCCGGGGCTACCAG	Ls.17, Ls.17-Δ*atg1*	Ls.17-Δatg1, PH-Δatg1
Vdatg1R	CTCGCCCGGCCAAGACAG		
**PCR validation of heterokaryons**			
hph-F	GGAGGGCGTGGATATGTCC	specific to the cassette conferring resistance to hygB	double-resistant heterokaryons (hygB^R and G418^R) obtained from the mixed infection experiments (*in planta*)
hph-R	CAGCTCATCGAGAGCCTG		
gen-F	GCTATGACTGGGCACAAC	specific to the cassette conferring resistance to G418	
gen-R	CAAGACCGGCTTCCATCC		
